# ORFannotate: reproducible coding sequence annotation of transcriptome assemblies

**DOI:** 10.1093/bioinformatics/btag082

**Published:** 2026-02-17

**Authors:** Sonia García-Ruiz, Hannah Macpherson, Laura Caton, Mina Ryten, Emil K Gustavsson

**Affiliations:** UK Dementia Research Institute, University of Cambridge, Cambridge, UK; Department of Clinical Neurosciences, School of Clinical Medicine, University of Cambridge, Cambridge, UK; Department of Neurodegenerative Disease, Queen Square Institute of Neurology, UCL, London, UK; Department of Neurodegenerative Disease, Queen Square Institute of Neurology, UCL, London, UK; Department of Genetics and Genomic Medicine, Great Ormond Street Institute of Child Health, University College London, London, UK; Department of Neurodegenerative Disease, Queen Square Institute of Neurology, UCL, London, UK; UK Dementia Research Institute at UCL, London, UK; UK Dementia Research Institute, University of Cambridge, Cambridge, UK; Department of Clinical Neurosciences, School of Clinical Medicine, University of Cambridge, Cambridge, UK; Department of Genomic Medicine, School of Clinical Medicine, The University of Cambridge, Cambridge, UK; UK Dementia Research Institute, University of Cambridge, Cambridge, UK; Department of Clinical Neurosciences, School of Clinical Medicine, University of Cambridge, Cambridge, UK; Department of Genetics and Genomic Medicine, Great Ormond Street Institute of Child Health, University College London, London, UK

## Abstract

**Summary:**

Accurate annotation of coding sequences and translational features within transcript models is essential for interpreting assembled transcriptomes and their functional potential. Existing open reading frame (ORF) prediction tools typically operate on transcript FASTA files and do not reintegrate coding sequence (CDS) information back into transcript models, limiting their utility in long-read sequencing workflows where GTF/GFF annotations are the primary output. We present ORFannotate, a lightweight, GTF-native Python command-line tool that predicts ORFs from transcript annotations and reinserts precise, exon-aware CDS and UTR features into the original GTF/GFF file. In addition, ORFannotate provides biologically informative translational context by annotating Kozak sequence strength, detecting non-overlapping upstream ORFs (uORFs) with coding probabilities, characterising 5′ and 3′ untranslated regions (UTRs), and predicting nonsense-mediated decay (NMD) susceptibility. All annotations are consolidated in a transcript-level summary to support downstream analysis. By generating GTF files with accurate CDS annotations, ORFannotate facilitates reproducible analysis of both long- and short-read transcriptomes and integrates seamlessly with visualization tools, genome browsers, and comparative transcript analysis workflows. ORFannotate is fast, scalable and provides a practical solution for transcriptome annotation beyond coding potential prediction alone.

**Availability and implementation:**

ORFannotate is implemented in Python and freely available under the GNU General Public License v3 (GPL-3.0) at: https://github.com/egustavsson/ORFannotate (DOI: https://doi.org/10.5281/zenodo.16812866)

## 1 Introduction

Alternative splicing is a key mechanism that expands transcriptomic and proteomic diversity by enabling a single gene to produce multiple distinct mRNA transcripts (Pan *et al.* 2008, Nilsen and Graveley 2010). These transcripts can differ in stability, localization, translational efficiency, and function (Kalsotra and Cooper 2011, Baralle and Giudice 2017). Accurate transcript identification is therefore essential for understanding gene function and regulation.

Long-read RNA sequencing technologies now provide the ability to sequence full-length transcripts without the need to infer transcript structures from short fragments, substantially increasing the resolution with which transcript diversity can be characterised. As long-read sequencing expands the catalogue of known and novel transcripts, there is a corresponding need for tools that can accurately interpret and annotate this complexity. A key aspect is to understand whether a transcript is likely to be translated into a protein.

Several methods exist for predicting open reading frames (ORFs) or coding potential from transcript sequences, including CPAT (Wang *et al.* 2013), TD2 (Mao *et al.* 2025), and ORFfinder; however, these methods typically operate on transcript FASTA input and do not integrate directly with GTF/GFF annotations. Other approaches, such as TransDecoder, require users to convert transcript models to FASTA sequences and, after ORF prediction, remap ORF coordinates back to the genome using additional utilities. Reference-guided tools such as ORFanage can assign ORFs with high accuracy for well-annotated loci; however, they do not modify the original GTF/GFF file to include coding sequence (CDS) features, and their reliance on reference annotation makes them less suited to settings where many transcripts are novel or structurally diverse, which are frequently uncovered in long-read datasets. In addition, existing ORF prediction tools typically focus on identifying a coding sequence but do not provide a consolidated summary of translational features such as Kozak context, upstream ORFs (uORFs), UTR structure, or predicted susceptibility to nonsense-mediated decay.

Here we present ORFannotate, a lightweight Python-based tool that directly addresses these gaps by predicting coding ORFs from transcript annotations and enriching them with biologically informative translational context. ORFannotate reconstructs full-length transcript sequences directly from exon coordinates, identifies the highest-scoring ORF per transcript using CPAT and reinserts the corresponding exon-aware CDS, together with UTR features, into the original GTF. ORFannotate also outputs the top *N* predicted ORFs per transcript in FASTA format, each containing the ORF sequence and its CPAT coding probability, allowing users to inspect alternative ORFs beyond the primary annotated CDS. By producing exon-aware CDS and UTR annotations, ORFannotate ensures convenient compatibility with downstream visualization and comparative analysis tools, such as ggtranscript, that depend on precise CDS information to accurately render transcript structures (Gustavsson *et al.* 2022).

Beyond CDS annotation, ORFannotate provides transcript-level metrics that help assess the translational potential and regulatory properties of each transcript. These include Kozak sequence strength, which can influence translation initiation efficiency; non-overlapping upstream ORFs (uORFs) with their coding probabilities, which may modulate translation of the downstream CDS; and predictions of nonsense-mediated decay (NMD) using the 50–55 nt rule, providing insight into transcript stability. ORFannotate also characterises 5′ and 3′ UTRs by their lengths and exon–exon junction counts and inserts exon-aware UTR features into the GTF, enabling accurate visualisation of untranslated regions and their splicing patterns.

ORFannotate is designed to integrate seamlessly with transcriptome assembly tools, such as Bambu (Chen *et al.* 2023), FLAIR (Tang *et al.* 2020), IsoQuant (Prjibelski *et al.* 2023), StringTie2 (Kovaka *et al.* 2019), and TALON (Wyman *et al.* 2019), without conversion or preprocessing steps. These tools typically produce transcript models in GTF or GFF3 format, which ORFannotate can parse natively without conversion to FASTA. This flexibility enables seamless integration into a wide range of transcriptome analysis workflows without introducing format incompatibilities or preprocessing steps. Its minimal dependencies and modular architecture allow incorporation into diverse annotation workflows, including SQANTI3 (Pardo-Palacios *et al.* 2024). While tools such as SQANTI3 offer comprehensive QC and annotation frameworks for long-read transcriptomes, these are often tightly coupled with specific pipelines and scoring methods and may be less flexible in modular workflows. In contrast, the modular compartmentalisation of ORFannotate offers flexible customisation of any of its major features, including ORF scoring, exon-aware CDS generation, and transcript-level summaries such as UTR junction count, Kozak context, uORF information and NMD predictions. This makes ORFannotate a GTF-native tool seamlessly integrable into a wide range of transcriptome analysis workflows without introducing format incompatibilities or preprocessing steps.

## 2 Implementation

ORFannotate is implemented in Python and performs transcript-level ORF and UTR annotation using standard GTF and genome FASTA inputs. The tool is freely available at https://github.com/egustavsson/ORFannotate (DOI: https://doi.org/10.5281/zenodo.16812866) and is distributed with a Conda environment file to streamline installation and ensure reproducible execution. Users can run the pipeline directly from source with minimal input. Full instructions, example datasets, and output descriptions are available in the online documentation, and key parameters can be modified via the configuration file (config.json).

ORFannotate requires three inputs: (i) a transcriptome file in GTF or GFF3 format, (ii) a genome reference FASTA file, and (iii) selection of one of the bundled CPAT models (human, mouse, fly, zebrafish). A custom CPAT model can be supplied for other organisms or retrained classifiers.

**Figure 1:**
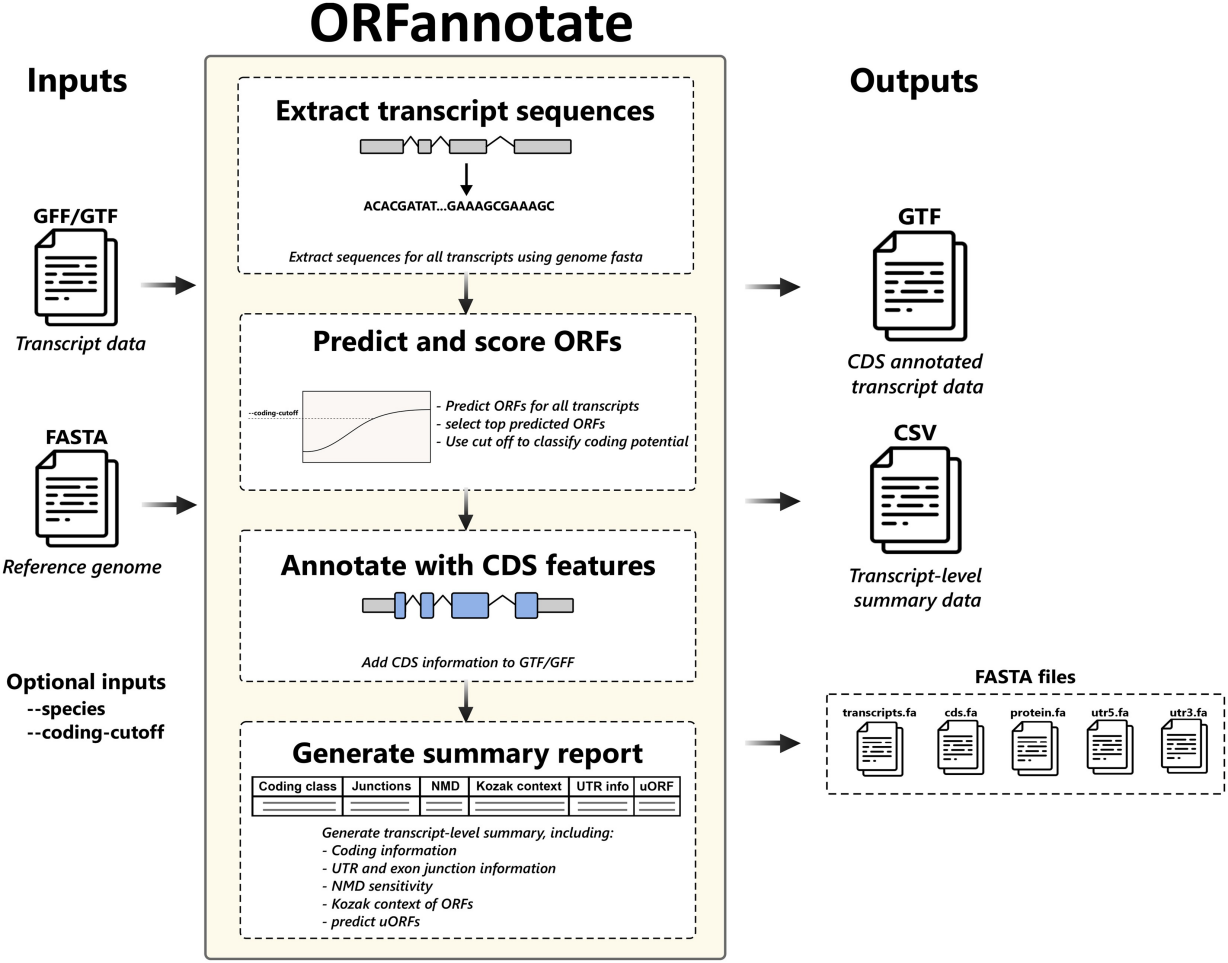
Schematic illustration of ORFannotate, a Python-based tool for predicting coding open reading frames (ORFs) from transcript annotations (GTF/GFF). ORFannotate identifies and prioritises candidate coding ORFs, inserts accurate, junction-aware CDS features into annotation files, and generates transcript-level summaries alongside FASTA outputs for CDS, protein, and UTR sequences. The tool supports configurable ORF selection criteria and detection of upstream ORFs (uORFs) within 5′ untranslated regions.

ORFannotate proceeds through seven main steps (Fig. 1). First, transcript sequences are reconstructed from exon coordinates using the reference genome. Second, up to *N* in-frame ORFs beginning with an AUG codon are predicted and scored using CPAT (default *N *= 5). The ORF with the highest coding probability is selected as the primary ORF and is used for all downstream annotation. The remaining predicted ORFs are stored together with their sequences and CPAT coding probabilities, and the value of *N* can be modified via the configuration file. A CPAT coding-probability threshold is then applied to classify transcripts as coding or non-coding, with recommended species-specific cut-offs provided by default unless overridden by the user.

Third, for transcripts classified as coding, the selected ORF is mapped back to the transcript structure to generate exon-aware CDS features, which are inserted into the original GTF. Reinserting CDS features ensures immediate compatibility with genome browsers and visualisation tools, enabling intuitive interpretation of transcript structures. Fourth, if the best ORF has a potential uORF that does not overlap with the selected ORF and its coding probability is above the minimum cut-off, this information is retained and incorporated into the final summary output together with its coding potential. Fifth, the Kozak sequence surrounding the start codon (positions −6 to +4) is scored and classified as strong, moderate, or weak based on the −3 and +4 positions. Sixth, a simple heuristic for NMD is applied: transcripts with a stop codon more than 50 nucleotides upstream of the final exon–exon junction are flagged as NMD-sensitive. Seventh, the number of 5′ and 3′ UTR exon-exon junctions is provided and exon-aware UTR features are injected into the original GTF.

Finally, ORFannotate generates a transcript-level summary table containing the ORF coordinates, coding probability, coding classification, Kozak score, NMD flag, UTR lengths, UTR exon-exon junction count, uORF information, and transcript biotype. FASTA files for the coding sequence (CDS), translated protein sequence, and 5′/3′ UTRs are exported for all coding transcripts.

ORFannotate is executed via the ORFannotate.py script, which integrates modular components for each processing step and organizes all intermediate files, logs, and outputs in a user-defined directory. This modular structure facilitates maintenance, debugging, and the addition of new features in future versions, allowing individual steps to be updated or extended without rewriting the entire pipeline. The tool is optimized for large-scale transcriptome datasets and exhibits efficient runtime performance.

Benchmarking was performed on two separate high-performance computing (HPC) clusters to illustrate scalability and provide users with a practical indication of expected runtime and memory requirements. The first test was run in an HPC node running Ubuntu 22.04.3 LTS (Jammy Jellyfish) equipped with two AMD EPYC 7742 64-Core Processors (256 hardware threads). To ensure reproducibility and fair scaling comparisons, all runs were restricted to a single CPU core by setting environment variables OMP_NUM_THREADS = 1 MKL_NUM_THREADS = 1 OPENBLAS_NUM_THREADS = 1. The second test was performed in a single node within an HPC environment running Rocky Linux release 8.10 (Green Obsidian) equipped with an Intel(R) Xeon(R) Platinum 8276 CPU @ 2.20 GHz, and 15GiB of allocated memory.

Under these single-threaded conditions, runtime and memory usage scaled almost perfectly linearly with dataset size: runs of 10, 100, 1000, 10 000, 50 000, 100 000, 200 000 and 385 669 transcripts took approximately 5 s, 5 s, 11 s, 37 s, 3.5 min, 6.3 min, 12.0 min, and 24.6 min, respectively, while peak memory usage increased from 85 MB to 6.2 GB. During the second test, ORFannotate completed the processing of 385 669 (Homo_sapiens.GRCh38.114.chr.gtf) and 506 944 (Homo_sapiens.GRCh38.115.chr.gff3) unique transcript structures in 22.4 and 36.6 min, respectively. Peak memory usage corresponded to 4.75 GB and 9.68 GB in each case. ORFannotate is freely available under the GPLv3 license and is distributed with pre-trained CPAT models, example datasets, and comprehensive documentation to facilitate reproducible analyses.

## 3 Discussion

The increasing use of long-read RNA sequencing has transformed our ability to identify and characterise full-length transcript isoforms. Although many transcriptome assembly and classification tools provide detailed structural annotations and compare assembled models to reference gene sets, they often lack a standardised step for annotating coding sequences and related translational features. ORFannotate fills this gap by working directly on GTF or GFF3 files to predict coding potential using CPAT, insert exon-aware CDS and UTR features, and generate a comprehensive per-transcript summary including Kozak context, uORFs, and NMD predictions.

ORFannotate is designed to operate downstream of transcriptome assembly, ensuring broad compatibility with common tools and avoiding the need for intermediate FASTA conversions. In this way, ORFannotate provides a lightweight yet informative annotation layer that can be readily incorporated into downstream analyses, visualisation tools, and functional interpretation pipelines. In this way, it complements broader annotation frameworks such as SQANTI3 by focusing specifically on ORF, CDS, UTR, and translational-context annotation in a GTF-native manner.

ORFannotate is fast, simple to use, and scalable to hundreds of thousands of transcripts, making it suitable for both exploratory analyses and large-scale, multi-sample transcriptomics projects. While the current version selects a single primary ORF for insertion into the GTF, additional high-scoring ORFs are retained for inspection, enabling users to explore alternative coding possibilities where relevant. Future releases could extend ORFannotate’s capabilities by incorporating additional evidence layers, such as ribosome profiling, or proteomics, and by offering more flexible strategies for representing alternative ORFs within the GTF output.

In summary, ORFannotate provides a practical and interoperable solution for post-assembly ORF and UTR annotation, enabling reproducible, interpretable, and efficient analysis of transcriptomes generated using both long- and short-read sequencing technologies. By adding biologically informative translational features and producing GTF-native outputs, ORFannotate complements existing long-read analysis frameworks and supports a wide range of downstream transcriptomics applications.

## Data Availability

No new data were generated or analysed in support of this research.
